# Choosing Wisely—Barriers and Solutions to Implementation in Low and Middle-Income Countries

**DOI:** 10.3390/curroncol29070403

**Published:** 2022-07-19

**Authors:** Fidel Rubagumya, Manju Sengar, Sidy Ka, Nazik Hammad, Christopher M. Booth, Safiya Karim

**Affiliations:** 1 Department of Clinical Oncology, Rwanda Military Hospital, Kigali 3377, Rwanda; 2 Division of Cancer Care and Epidemiology, Queen’s University Cancer Research Institute, Kingston, ON K7L 3N6, Canada; christopher.booth@kingstonhsc.ca; 3 Departments of Oncology, Queen’s University, Kingston, ON K7L 3N6, Canada; nazik.hammad@kingstonhsc.ca; 4 Tata Memorial Centre, Homi Bhabha National Institute, Mumbai 400094, India; manju.sengar@gmail.com; 5 Cheikh Anta Diop University, Dakar 5005, Senegal; kasidyoasis@gmail.com; 6 Tom Baker Cancer Centre, University of Calgary, Calgary, AB T2N 1N4, Canada; safiya.karim@albertahealthservices.ca

**Keywords:** Choosing Wisely, Africa, value-based cancer care, low and middle-income countries

## Abstract

Globally, there is increasing emphasis on value-based cancer care. Rising healthcare costs and reduced health care spending and budgets, especially in low- and middle-income countries (LMICs), call for patients, providers, and healthcare systems to apply the Choose Wisely (CW) approach. This approach seeks to advance a dialogue on avoiding unnecessary medical tests, treatments, and procedures. Several factors have been described as barriers and facilitators to the implementation of the Choosing Wisely recommendations in high-income countries but none for LMICs. In this review, we attempt to classify potential barriers to the Choose Wisely implementation relative to the sources of behavior and potential intervention functions that can be implemented in order to reduce these barriers.

## 1. Introduction

Globally, there is increasing emphasis on value-based cancer care [1]. Rising healthcare costs reduced healthcare spending and budgets. Recent evidence shows that overuse in cancer-care delivery has caused patients, providers, and healthcare systems to carefully examine their practices [2]. Value-based cancer care advocates for diagnostic and therapeutic interventions that are backed by evidence for safety and efficacy as well as cost-effectiveness and discourages the delivery of ineffective, unproven, harmful, or low-value practices, treatments, programs, and interventions [3]. Value-based care delivery is relevant for all health systems, but it is of paramount importance in low and middle-income countries (LMICs) given their limited health budgets, inadequate health infrastructure, and the double burden of disease, among others [4]. The rising burden of cancer in LMICs is stretching the already-fragile health care system. Individually, the costs of cancer treatment may lead to catastrophic or impoverished personal health care expenditures [5]. Hence, these low-value practices result in the diversion of resources and, thus, have a detrimental effect on overall outcomes in resource-limited settings.

Standard treatment guidelines, including resource-stratified guidelines for cancer, are available to inform cancer care in LMICs and are one of the method for disseminating the practice of value-based care [6,7,8,9]. However, these guidelines alone do not prevent low-value practices.

## 2. Choosing Wisely Initiative

One initiative that promotes value-based cancer care is Choosing Wisely (CW). This campaign seeks to advance a dialogue on avoiding unnecessary medical tests, treatments, and procedures [10]. CW Oncology originated in the United States (2010), and it was later adopted in Canada (2014) [10,11]. Globally, CW Oncology now has campaigns in India [4] and Africa [4,12]. Each campaign lists practices that physicians should question to avoid unnecessary care in the treatment of patients with cancer. Some listed practices are similar between countries while others are country- or region-specific given differences in many factors including infrastructures and health-care delivery systems.

While the CW campaign has gained significant momentum over the past years, research has shown that the publication of the list alone is insufficient for impacting clinical practice [13,14,15]. Several studies have shown that concordance to CW guidelines is suboptimal [16,17]. A major reason for the poor penetration of CW guidelines in clinical practice is a lack of awareness among practitioners. While there are no studies that evaluate the awareness about CW guidelines among clinicians, it is unlikely that it will be different from awareness about other practical guidelines, as a recent study exploring the awareness of contouring guidelines among radiation oncologists in US reported that only 42% were aware of the these guidelines [13]. Given this low level of awareness among frontline clinicians, it is not surprising that the CW guidelines have been sub-optimally implemented. Furthermore, despite the enthusiasm to spread CW campaigns globally and in different disciplines, there has been little research conducted for evaluating the best implementation strategy, especially in LMICs.

## 3. Frameworks for Implementing CW

Several factors have been described as barriers and facilitators to implementing clinical guidelines, and these can also be applied to the CW recommendations. Given contextual differences, barriers and facilitators with respect to adopting CW recommendations will be different in LMICs compared to high-income countries (HICs). For example, compared to HICs, patients and care providers in LMICs are less likely to be aware of CW initiatives, and most leave decision making about their care to physicians [18,19].

Several frameworks for implementing value-based cancer care and de-implementing low-value practices have been proposed [3,20]. Norton et al. proposed a framework that can be used to de-implement cancer clinical practice. This framework includes five factors: strength of evidence, magnitude of the problem, action, barriers and facilitators, and strategy [3]. Factors such as the strength of evidence, magnitude of the problem, and action can be considered as guiding principles when developing an initial list of CW recommendations. Within the barriers/facilitators and strategies factor, the authors propose that this needs to be considered from the patient, provider, healthcare delivery setting, and societal perspective. These factors can also be expanded; for example, provider factors can include knowledge and attitude, guideline factors such as format and content, and external factors such as a lack of resources, organizational constraints, heavy workload, society, and cultural norms among others [21,22].

The implementation of CW recommendations in LMICs requires an understanding of specific barriers and behaviour change interventions that can address these barriers. Michie’s capability, opportunity, and motivation behaviour (COM-B) system and the behaviour change wheel (BCW) are frameworks that are commonly used [23].

The COM-B model has been used as a framework for addressing barriers with respect to the implementation of various guidelines in other disciplines, especially in high-income countries [24,25,26]. In LMICs, De Boer et al. used the COM-B framework to identify barriers with respect to the implementation of Tanzania’s first National Treatment Guidelines. They also used the behaviour change wheel (BCW) framework to select interventions to address each barrier and behavior change techniques to enact each intervention function and a mode of delivery [22]. 

Using Norton’s analytical framework, the COM-B model, and the BCW, we attempt to classify potential barriers to CW implementation into the sources of behaviour and potential intervention functions that can be implemented (Table 1) [23].

## 4. Discussion

Using socio-ecological frameworks, barriers to the implementation of CW recommendations can be categorized into patient-related, provider-related, health system-related, and society-related. Patient barriers a include lack of awareness, limited acceptability, and a lack of trust between patients and physicians, among others. These can be linked to capability where the patient does not possess the knowledge or skills to understand why a particular practice is unnecessary.

Educational initiatives such as making the CW recommendations easy to understand from a patient perspective and raising awareness of the campaign amongst patients are potential methods for mitigating these barriers. Provider-related barriers include a lack of awareness, fear of litigation, and other competing clinical guidelines. Provider-specific training sessions, identifying champions of the CW recommendations, and making the CW list visible in clinics/hospital wards can be considered. Health system-related barriers include a lack of leadership support, accountability, and incentives to use unnecessary practices for revenue generation. Environmental restructuring and education initiatives targeted at individuals in upper management and leadership positions can be used to address these barriers. Specifically, it is essential to emphasize to hospital leadership that value-based cancer care benefits patients and healthcare systems, including financially. De-incentivizing low-value practices at the hospital level can also include reducing public funding and removal from insurance providers. Models that reflect the financial impact of low-value interventions should be encouraged. These can show the financial loss at the patient level with respect to individual’s earnings and at the society level, assuming that the lost funding would have been distributed to other areas. Finally, society-related barriers include cultural norms, regulations, and health policy. Strategies to mitigate barriers at the societal level are arguably the hardest to implement but may also have the most significant impact. These can include mass media campaigns, working with regulators to advertise warnings for low-value drugs or interventions, and working with insurance providers to prioritize reimbursement of value-based care. 

## 5. Conclusions and Future Directions

The CW campaign can be one of the most impactful initiatives in oncology to improve health services across the countries. However, ground-level implementation requires concerted efforts at multiple levels, including patient, provider, health system, and societal levels. It is vital to address each of the stated barriers in a multi-step manner with a quantitative impact analysis, which will serve as positive feedback for all the stakeholders.

## Figures and Tables

**Table 1:** COM-B Model and Barriers to Implementation of the CW Recommendations.

Barriers	COM-B Category	Intervention Functions	Behavior Change Techniques and Mode of Delivery
**Patient-related**			
Lack of patient awareness of the CW recommendations	Physical Capability	Education Enablement	Translate CW recommendations in an easy to understand language and distribute in waiting areas
Lack of trust between patient and providers	Psychological Capability	Education	Carry out campaigns encouraging patients to talk with their provider
Limited acceptability	Psychological Capability	Education	Create culturally tailored learning modules for patients
Patients beliefs that more is better	Psychological Capability	Education Persuasion	Educate patients on the problem of overuse
**Provider-related**			
CW recommendations/list not easily accessible	Physical Capability	Enablement	Distribute CW list hard copies to every unit and clinic room and soft copies to every provider
Providers not knowledgeable of CW list	Psychological Capability	Education	Teach CW content, including evidence basis for these recommendations to providers in dedicated education session and integrate into existing curriculum for residents and nurses.
Providers do not believe that they should be following CW recommendations	Psychological Capability	Education Persuasion Modeling	Publicity campaign to raise awareness of the CW initiative. Choose local ‘Implementation Champions’ who will persuade providers that they should adhere to CW recommendation, and model this behavior during morning conferences and in clinical practice.
Belief that expertise-based decisions are better than CW recommendations	Reflective Motivation	Training Persuasion	Train providers in the benefits of CW-based practice and persuade them that they should be used in favor of expert opinion.
Fear of Litigation	Psychology Capability	Education	Carry out workshops and show that the CW list is based on evidence; hence, providers should not fear litigation.
**Health System-related**			
Lack of leadership support	Physical Opportunity	Environmental Restructuring	Engage hospital leadership and show them the benefit of value-based cancer care
Revenue generation –Reluctance to implement CW recommendations as this may lead to reduced revenues	Physical Opportunity	Environmental Restructuring	Engage hospital leadership and show detrimental effects of overuse for both patients and the hospitals. Provide examples of other health care systems that have prioritized value-based care without financial detriment.
Lack of accountability in patient management	Physical Opportunity	Education Environmental Restructuring	Encourage hospitals to carry out audits and provide formal feedback
**Society-related**			
Cultural Norms	Psychological Capability	Education Persuasion	Mass media campaigns
Regulations	Social Opportunity	Training Modeling	Health regulator product warnings
Health Policy	Physical Opportunity	Education Environmental Restructuring	Introduction of value-based reimbursement policies amongst insurance providers.

From Ref. [22], used under the terms of the Creative Commons Attribution 4.0 International License (http://creativecommons.org/licenses/by/4.0/ (Accessed on 15 July 2022).
